# Zooplankton as a Transitional Host for *Escherichia coli* in Freshwater

**DOI:** 10.1128/aem.02522-21

**Published:** 2022-04-13

**Authors:** Andrea Di Cesare, Francesco Riva, Noemi Colinas, Giulia Borgomaneiro, Sara Borin, Pedro J. Cabello-Yeves, Claudia Canale, Nicholas Cedraro, Barbara Citterio, Elena Crotti, Gianmarco Mangiaterra, Francesca Mapelli, Vincenzo Mondino, Carla Vignaroli, Walter Quaranta, Gianluca Corno, Diego Fontaneto, Ester M. Eckert

**Affiliations:** a Molecular Ecology Group, National Research Council of Italy, Water Research Institute (CNR-IRSA) Verbania, Italy; b Department of Food, Environmental and Nutritional Sciences, University of Milan, Milan, Italy; c Institut Cavanilles de Biodiversitat i Biologia Evolutiva, Universitat de València, Valencia, Spain; d Evolutionary Genomics Group, Departamento de Producción Vegetal y Microbiología, Universidad Miguel Hernández de Elche, San Juan de Alicante, Alicante, Spain; e Microbiology Lab, ASL-VCO Castelli Hospital, Verbania, Italy; f Department of Life and Environmental Sciences, Polytechnic University of Marche, Ancona, Italy; g Department of Biomolecular Sciences, Biotechnology Section, University of Urbino “Carlo Bo”, Italy; h Tropical and Infectious Diseases Unit, ASL-VCO Castelli Hospital, Verbania, Italy; Royal Netherlands Institute for Sea Research

**Keywords:** *Escherichia coli*, *Daphnia*, fecal indicator bacteria, freshwater, lake, zooplankton

## Abstract

This study shows that Escherichia coli can be temporarily enriched in zooplankton under natural conditions and that these bacteria can belong to different phylogroups and sequence types (STs), including environmental, clinical, and animal isolates. We isolated 10 E. coli strains and sequenced the genomes of two of them. Phylogenetically, the two isolates were closer to strains isolated from poultry meat than to freshwater E. coli, albeit their genomes were smaller than those of the poultry isolates. After isolation and fluorescent protein tagging of strains ED1 and ED157, we show that *Daphnia* sp. can take up these strains and release them alive again, thus becoming a temporary host for E. coli. In a chemostat experiment, we show that this association does not prolong bacterial long-term survival, but at low abundances it also does not significantly reduce bacterial numbers. We demonstrate that E. coli does not belong to the core microbiota of *Daphnia*, suffers from competition by the natural *Daphnia* microbiota, but can profit from its carapax to survive in water. All in all, this study suggests that the association of E. coli with *Daphnia* is only temporary, but the cells are viable therein, and this might allow encounters with other bacteria for genetic exchange and potential genomic adaptation to the freshwater environment.

**IMPORTANCE** The contamination of freshwater with feces-derived bacteria is a major concern regarding drinking water acquisition and recreational activities. Ecological interactions promoting their persistence are still very scarcely studied. This study, which analyses the survival of E. coli in the presence of zooplankton, is thus of ecological and water safety relevance.

## INTRODUCTION

Fecal bacteria can enter aquatic environments by different routes, e.g., sewage discharge or direct fecal deposition (1). Although fecal bacteria are tendentially seen to rapidly drop in abundance once outside their host, some aquatic environments might allow their long-term survival and growth (1). Escherichia coli is a common member of the gut microbiota of vertebrates (2). Thus, it is commonly released by the fecal route into aquatic environments (3), and it is therefore used as a fecal indicator bacterium (FIB) to evaluate anthropogenic water pollution (4). Clinically relevant E. coli strains (5, 6), including antibiotic resistant isolates (7–9), can be found in water. Moreover, there is evidence that E. coli can adapt to a freshwater lifestyle, as shown through its differential gene expression once incubated in water (3).

Naturalized E. coli, e.g., E. coli that entered the aquatic environment from the gut and then adapted to this lifestyle, have been isolated from lake sediments and phytoplankton, and different studies have shown the capability of this species to genetically adapt and persist in the environment (10–13). Several E. coli isolated from freshwater had a small genome size and other peculiarities at the genomic level which suggested an evolutionary adaptation to this habitat (14). This is particularly interesting since genome reduction has been repeatedly proposed as an adaptation to aquatic environments in common environmental bacteria (15, 16) and in experimental systems (17, 18). Thus, an aquatic environment may contribute to the genetic evolution of E. coli (14).

However, mammal-associated fecal bacteria usually persist badly in cold habitats such as deep lakes. In particular, pelagic cold waters are a very hostile environment for such gut symbionts. If they are not grazed by flagellated predators upon arrival, they are easily outcompeted by bacteria which grow better at low nutrient concentrations (19–21). Furthermore, E. coli is a facultative anaerobe and grows best at low oxygen concentrations (22). Because evolution, as seen in the genome reduction of E. coli (14), takes time, a certain long-term persistence of vertebrate commensal strains in the aquatic habitat is crucial, and the question remains: in which niche does persistence take place? In clinical settings, these bacteria thrive better in biofilms (23), and might persist in a similar niche in aquatic habitats (24, 25). In lake environments, biofilms can be formed on dead organic and inorganic material, sediments, stones, and animals, and FIB have been found in sediments (26, 27), in macrophytes (28), and on fish (29, 30), for example. Much less attention has been devoted to small invertebrates, i.e., zooplankton, as potential hosts for these bacteria. Such animals are interesting since their microbiota seems to be composed of many transient microbes and thus is likely more prone to invasion by allochthonous bacteria (31–34). In fact, antibiotic-resistant bacteria were easily removed from the surrounding water in a laboratory experiment, but persisted in the crustacean *Daphnia obtusa* (35), and FIB have been shown to even exchange genetic material in Daphnia pulex (36). It is generally assumed that the presence of *Daphnia* sp. reduces E. coli abundance in the water (37, 38). Nevertheless, in a study based on 16S rRNA gene amplicon sequencing from a lake, E. coli*/Shigella* made up a large percentage of the copepod and *Daphnia* microbiota, but were present only at low abundance on stones, in water, and in sediments (39). In this study, therefore, we wanted to investigate the nature of the relationship between E. coli and *Daphnia* in the freshwater environment to clarify the possible role that *Daphnia* might have in the persistence of E. coli.

Here, we tested the hypothesis that an association with zooplankton of the genus *Daphnia* could help a FIB, E. coli, by providing a niche in a freshwater lake, since the surrounding water provides suboptimal growth conditions for E. coli (low nutrients and high oxygen) and a high risk of predation. It has been shown that, in a few hours, the presence of Daphnia pulex reduces the abundance of surrounding E. coli (37); but here, we were interested in the longer-term association of the bacterium with the animal under natural conditions. Our hypothesis is that such an association might help the bacterium to adapt to this environment. Therefore, we quantified *uidA*, an indicator gene of E. coli, in DNA extracted from various potential niches for FIB in a freshwater lake, including stone biofilms, zooplankton, and sediment, and compared it to the pelagic water. Moreover, we searched for the occurrence of E. coli-related 16S rRNA reads in a large data set of zooplankton-associated microbiota. We then isolated E. coli strains from a *Daphnia* host, genotyped them, then tagged two of the strains with fluorescent proteins and sequenced their genomes. This allowed us to conduct experiments on the associations of these strains with an invertebrate host. Our hypothesis was that, despite *Daphnia* grazing reducing the abundance of E. coli in the water, its presence would still allow for better survival of the FIB over a longer duration thanks to the short-term refuge of part of the population within its gut. Moreover, we speculate that such an association might, in the long term, help E. coli adapt to freshwater over physiological and/or genetic adaptations.

## RESULTS

### Abundance of *E. coli* in Lake Maggiore.

**FIG 1:**
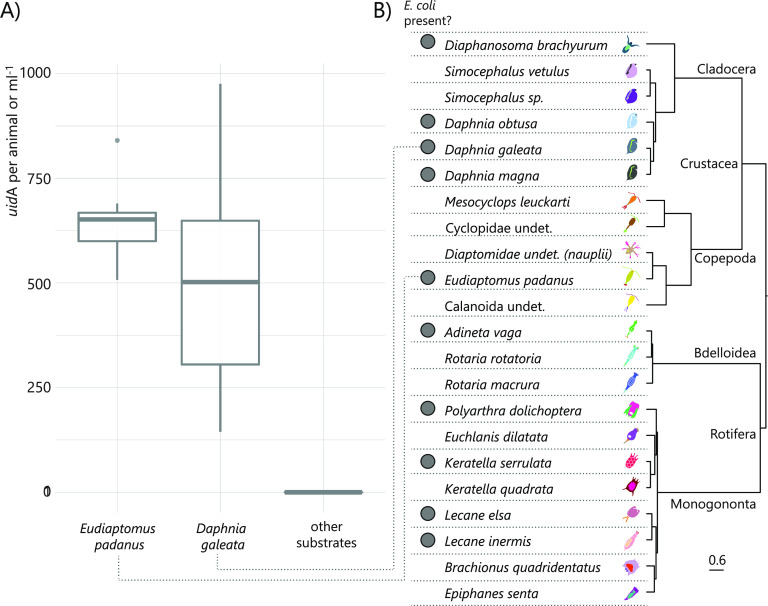
(A) Boxplots of the abundance of E. coli-specific *uidA* gene copies in DNA isolated from animals and other substrates that include sediments, stones, and water from 10- and 40-m depths from Lake Maggiore. For each plot, the thick horizontal line represents the median value, the box includes 50% of the data from the first to the third quartile, the whiskers extend to the minimum and maximum data within the 1.5 interquartile range, and the dots represent single outlier data points outside that range. (B) Occurrence of E. coli in various zooplankton species; a gray dot indicates that E. coli was found in the microbiome of at least one sample. Images and phylogeny of animals are shown as references and have been modified based on previous data (34).

By screening for the presence/abundance of the E. coli specific marker gene *uidA* in DNA extracted from three different locations in Lake Maggiore, we found that the gene was absent in sediments, epilithic biofilms, and water samples, but could be found in both *Daphnia* gr. *galeata/longispina* and copepods, showing between 144 and 976 (mean 580) copies per animal (Fig. 1A).

### E. coli in other zooplankton microbiomes.

We screened a large data set of zooplankton-related microbiomes and could find the presence of E. coli*/Shigella*-related 16S rRNA gene sequences in samples from other cladocerans (Daphnia magna, Daphnia obtusa, Diaphanosoma brachyurum*)* and rotifers (Adineta vaga, Keratella serrulata, Lecane elsa, Lecane inermis and *Polyarthra* sp.) (Fig. 1B). E. coli*/Shigella* was not found in other rotifers (Epiphanes senta, Keratella quadrata); Mesocyclops leukarti, a large calanoid copepod; or the cladoceran *Simocephalus* sp. (Fig. 1B). We quantified *uidA* in Daphnia obtusa sampled from a rainfed pond, because their E. coli*/Shigella*-related reads were particularly high; we also confirmed the presence of the E. coli *uidA* gene by qualitative real-time PCR.

### E. coli isolates.

**FIG 2:**
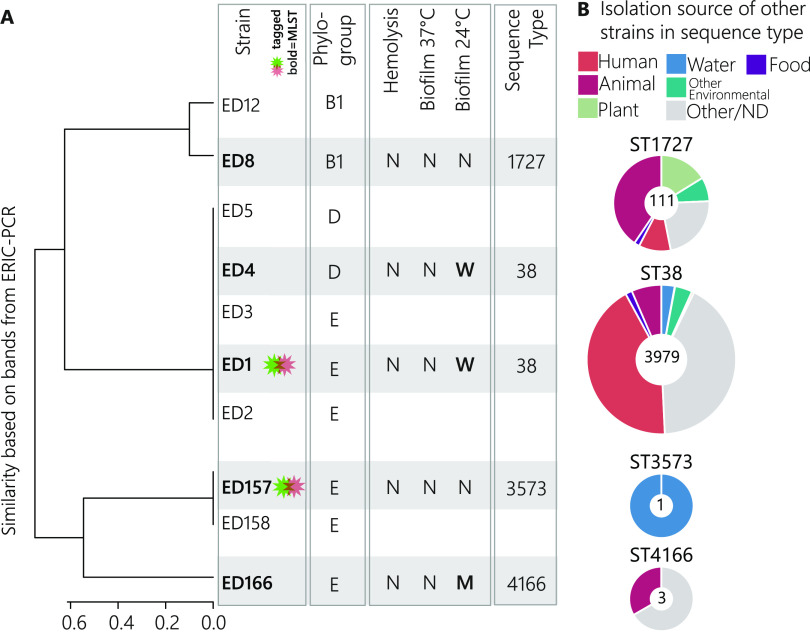
(A) Cluster dendrogram showing dissimilarity of ERIC profiles of the different E. coli strains isolated from *D. obtusa*, and their phylogroups according to the ERIC profile. Gray-shaded strains also presented data for their phenotype in the pathogenicity assay (N = none, M = medium, W = weak) and for their sequence type according to multi-locus sequence typing. (B) Pie charts summarizing the isolation sources of deposited E. coli strains of the same sequence type as the strains from daphnids; numbers at the middle of pie charts denote numbers of strains deposited for each sequence type.

**(i) Isolation of E. coli from *Daphnia obtusa*.** We attempted to isolate E. coli from *Daphnia obtusa* to further investigate which E. coli phylogroups were affiliated with zooplankton. Through multiple isolation campaigns, we retrieved 10 E. coli strains and identified their phylogroups: strains ED1, ED2, ED3, ED4, and ED5 formed one cluster and were affiliated with phylogroup D/E, and strains ED157, ED158, and ED166 formed a second cluster affiliated with phylogroup D/E (we did not succeed in discriminating between these two phylogroups for these strains), and strains ED8 and ED12 were affiliated with phylogroup B1 (Fig. 2A). Five of these strains were further chosen for multi-locus sequence typing (MLST) (ED1, ED4, ED8, ED157, and ED166) and pathogenicity assays: none of them showed traits of pathogenicity, except for weak biofilm formation in the isolates ED1, ED4, and ED166, and they were classified in four different sequence types (STs; ST38, ST1727, ST3573, and ST4166) (Fig. 2A and Table S2). We then analyzed the strains deposited in the MLST database affiliated with these STs and found that most of the ST38 isolates were of human origin, whereas ST1727 included more strains isolated from animals (41%) than from humans (10%). The other two STs (ST3573 and ST4166) have been rarely described and included isolates from nonhuman sources (Fig. 2B).

ED1 and ED157 were selected for further analysis. These two isolates were selected due to their STs: ED1 was affiliated with ST38, from which many other E. coli seem to be associated with mammals and some were even pathogens; whereas ED157 was affiliated with ST3573, which includes only one E. coli strain isolated from water. The genomes of ED1 and ED157 strains were sequenced, and the strains were successfully marked with green flourescent protein (GFP) and DsRed protein for interaction studies.

**FIG 3:**
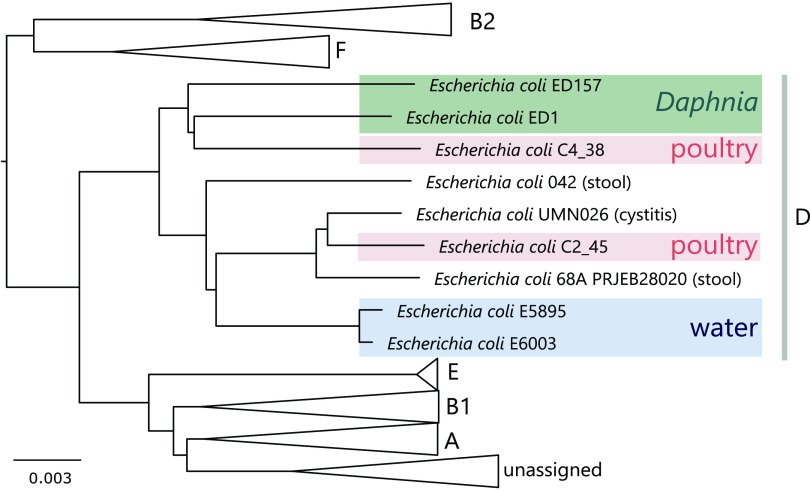
Phylogenetic tree of E. coli genomes included in Table 1. The tree was constructed using the MICROSCOPE tool with a neighbor-joining algorithm on Mash genomic distances. Scale bar represents 0.003 substitutions per nucleotide position.

**(ii) Genome analysis of ED1 and ED157.** Genome sequencing and analysis performed by ClermoTyper, and phylogenetic tree construction with MICROSCOPE, showed that the ED1 and ED157 genomes belong to the D phylotype (Fig. 3). Phylogenetic analysis performed on the whole-genome sequences of these strains, and the genomes of water- and poultry-isolated E. coli, showed that E. coli strains isolated from *Daphnia* sp. did not cluster with the water isolates. Conversely, the strains isolated from poultry meat clustered with the genomes of the *Daphnia* isolates, whereas the water-related E. coli clustered in a sister group (Fig. 3). When comparing genome sizes, we could observe that the ED1 and ED157 genomes were bigger than those of of strains isolated from water, but smaller than those of strains originating from poultry meat (Table 1). We found a higher number of plasmid replicon sequences in E. coli strains originating from poultry meat (C4_38, 5 plasmid replicon sequences; C2_45, 3 plasmid replicon sequences) than in those obtained from water (E5895, 1 plasmid replicon sequence; E6003, no plasmid replicon sequences detected) or from *Daphnia obtusa* (ED1, 2 plasmid replicon sequences; ED157, no plasmid replicon sequences detected) (Data Set S1 in the supplemental material). Poultry meat strains had more virulence genes (C4_38, 32 genes; C2_45, 29 genes) than the *Daphnia* (ED1, 12; ED157, 16) and water strains (E5895, 10; E6003, 12) (Data Set S1). Our analysis revealed that more phage sequences were present in the ED1 and ED157 genomes than in the other genomes analyzed. Specifically, we found 7 phage sequences in ED1 and 5 in ED157 (Data Set S1), whereas only 3 and 1 to 2 phage sequences were found in the poultry and water strains, respectively.

**TABLE 1:** Escherichia coli genomes included in the study, their lengths, their isolation sources, and the references where they were first reported

Strain	Accession no.	Total length (bp)	Isolation source	Reference
ED1	JAAWVB000000000	5,159,712	Daphnia obtusa	66
ED157	JABEXY000000000	5,273,211	Daphnia obtusa	This study
C4_38	ERS3883848	5,511,727	Poultry meat	14
C2_45	ERS3883832	5,623,389	Poultry meat	14
E5895	ERS3883463	4,825,729	Water	14
E6003	ERS3883339	4,771,985	Water	14

To compare which genes were different in the *Daphnia* isolates compared to other E. coli, a pangenomic analysis was performed with the strains listed in Table 1 using the protein family sorter tool of PATRIC (Table S3). The pangenome was composed of 7,108 protein families, while the core genome consisted of 3,789 protein families (53,7%). E. coli isolated from poultry meat and from *Daphnia* shared 57.5% protein families (from a total of 6,787), while E. coli isolated from *Daphnia* and from freshwater bodies shared 65% (from a total of 5,993).

The genomes from all groups shared a very high number of protein families related to habitat adaptation: for instance, we found the presence of protein families related to the production of capsular polysaccharides or to the type I fimbriae system. Interestingly, we detected the presence of RhS protein families, which are supposed to inhibit intercellular growth as their primary function (40), and of some protein families linked to sucrose utilization only in isolates from *Daphnia* and poultry meat. Focusing on the accessory genome of E. coli isolated from *Daphnia* (i.e., protein families that were not found in the other two groups), specific groups detected included xanthosine-related protein families, which allow bacteria to utilize purine nucleoside as a carbon and energy source; and poly-beta-1,6-*N-*acetyl*-*d*-*glucosamine (PGA) protein families, which are involved in the synthesis, export, and localization of PGA polymer, a necessary component for biofilm formation.

### Interaction of *E. coli* with *Daphnia obtusa*.

**FIG 4:**
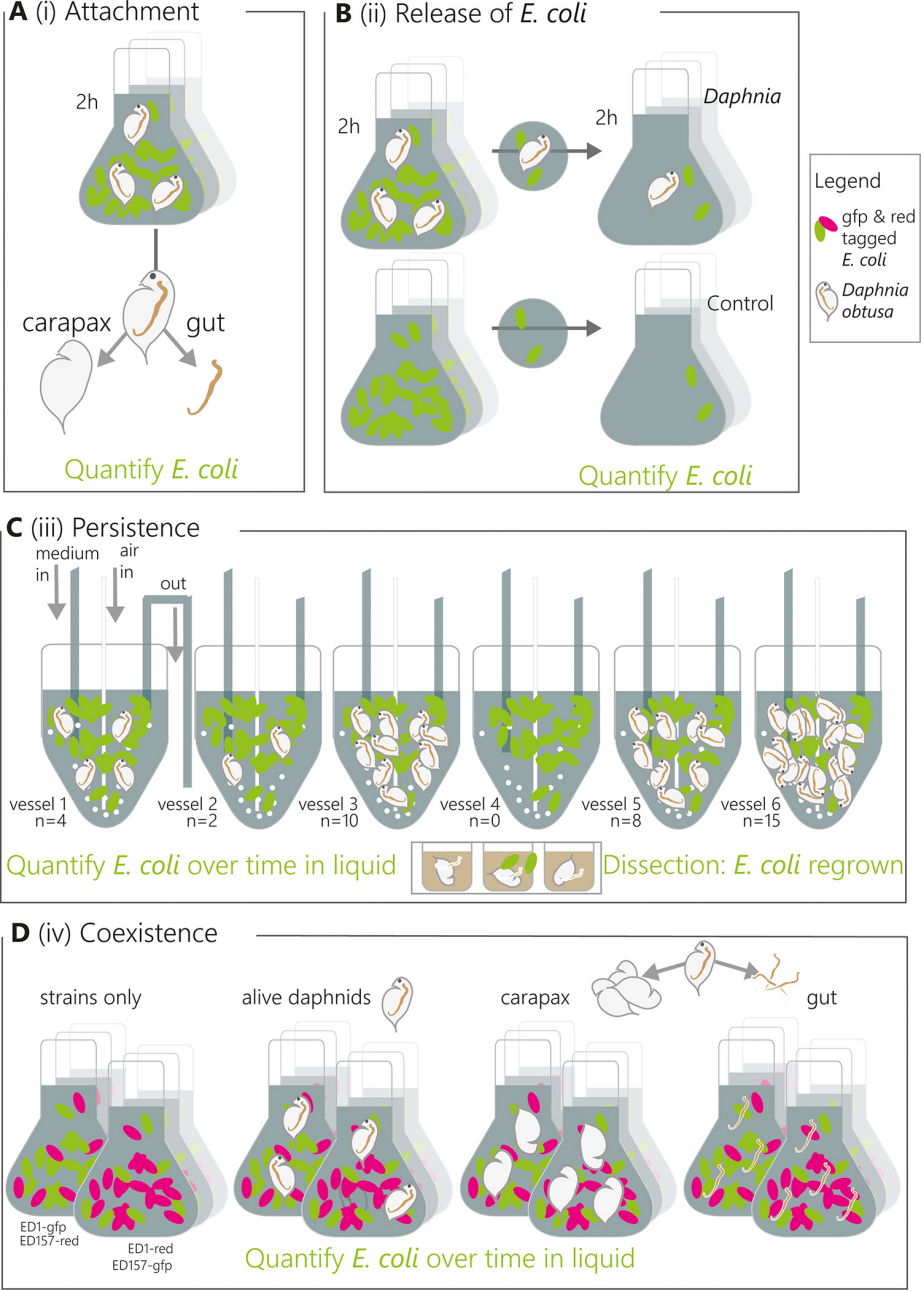
Experimental setup of all experiments involving E. coli and *Daphnia obtusa* association. (A to D) Setup of each experiment, with each panel corresponding to the same-titled subsection within Materials and Methods. (A) Localization of E. coli on *Daphnia*. (B) Release of E. coli after gut passage. (C) Persistence of E. coli with *Daphnia*. (D) Coexistence experiment. Experiments in panels A, B, and D were conducted in batches, while experiment C was conducted in a chemostat.

**(i) Attachment.** First, we verified where E. coli was localized in the animal by incubating ED1-gfp and ED157-gfp strains separately with live *Daphnia* for 4 h, dissecting the animals and performing a quantitative PCR (qPCR) assay targeting the *gfp* gene to verify the presence of E. coli on various body parts (Fig. 4A). We found that 70% ± 8% of the administered ED1-gfp and ED157-gfp were found in the gut compared to that in filter apparatus and carapax (data in Table S4).

**FIG 5:**
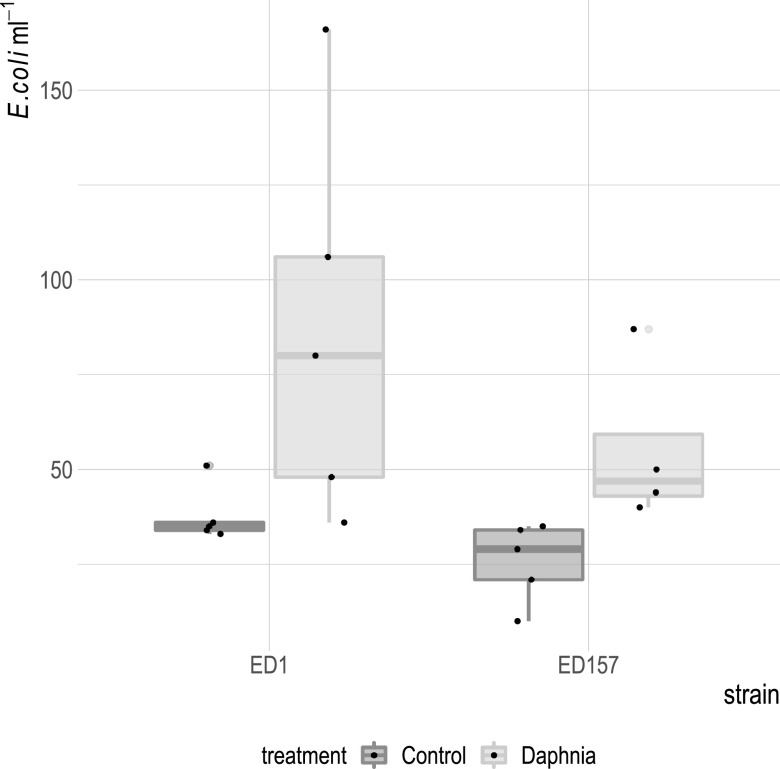
E. coli density in the treatments where only water was transferred (Control) and in treatments where animals (*Daphnia*) were transferred to sterile water after feeding on E. coli strains ED1-gfp (dark gray) or ED157-gfp (light gray). For each plot, the thick horizontal line represents the median value, the box includes 50% of the data from the first to the third quartile, the whiskers extend to the minimum and maximum data within the 1.5 interquartile range, and the gray dots represent single outlier data points outside that range. Original data points for each treatment are superimposed on the plots as black dots.

**(ii) Release.** We then tested whether E. coli was digested by *Daphnia*, whether *Daphnia* functioned as a refuge for E. coli, or whether E. coli simply passed through the gut. We incubated ED1-gfp and ED157-gfp with *Daphnia* for 4 h and then transferred them to new clean water (Fig. 4B). Compared to the control (transferred water without *Daphnia*), we found a significantly higher abundance of both E. coli strains in the surrounding water of the *Daphnia* group (Fig. 5; linear model ED157: estimate, 0.8 ± 0.3, *t* = 2.6, *P* = 0.03; ED1: estimate, 0.7 ± 0.3, *t* = 2.5, *P* = 0.03).

**FIG 6:**
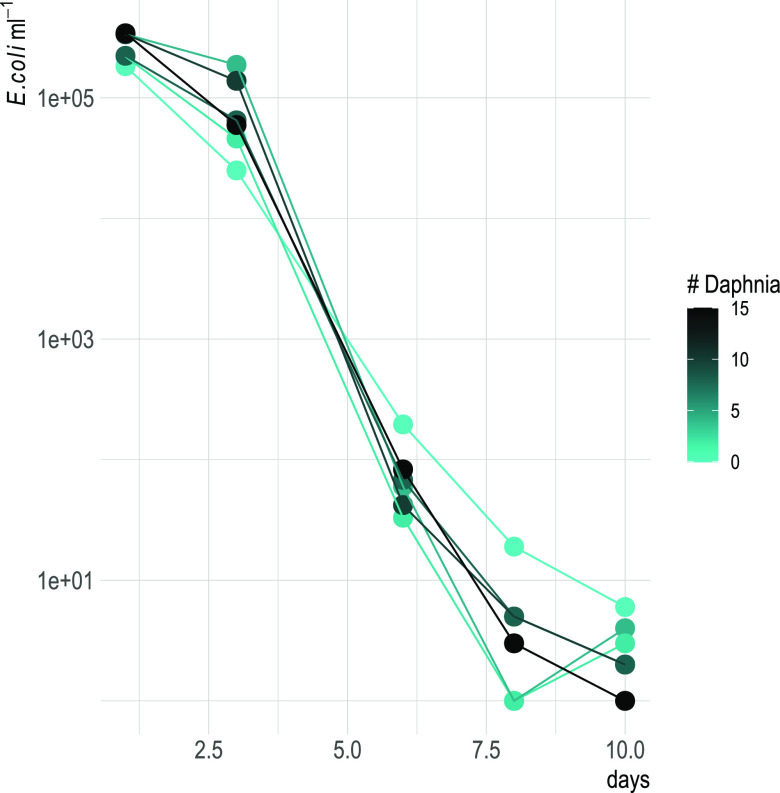
Cell density of tagged E. coli in chemostat experiment over time. Gradient values of the dots and lines indicate the starting numbers of *Daphnia obtusa* added to the vessels. *y* axis is log-scaled.

**(iii) Persistence with and without *Daphnia*.** We investigated how the presence of *Daphnia* impacted the general survival of ED1-gfp in freshwater systems in continuous culture experiments (chemostat). We therefore filled eight chemostat vessels with artificial lake water (ALW) medium, a phytoplankton culture, E. coli, and incubated *Daphnia* at different densities (0, 2, 4, 8, 10, 15 animals per vessel, Fig. 4C). We monitored the abundance of culturable E. coli in the water over time and found that the number of *Daphnia* had a slightly significant negative effect on the abundance of E. coli (*glm*: estimate = −0.11 ± 0.06, *z* = −2, *P* = 0.048). However, culturable E. coli abundances were in the same order of magnitude, with only 1 to 6 CFU detected per mL of surrounding water in all treatments after 10 days of incubation, even without animals (Fig. 6). Some animals were then washed and their guts dissected, and E. coli cells were regrown in a plate reader to see whether there were culturable E. coli cells in the guts of the animals. One-fourth of the 12 total dissected adult *Daphnia* (2 from vessel 5, 1 from vessel 3, 0 from vessels 1 and 6) displayed E. coli growth within the first 48 h of incubation, whereas no growth was detected in the guts of juvenile animals (total of 9 animals, Data Set S2).

**FIG 7:**
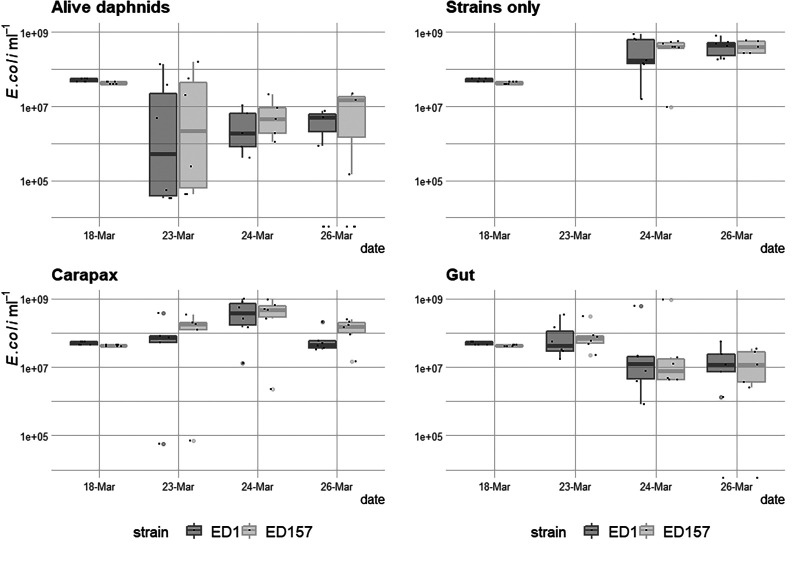
E. coli cell density over time in a batch experiment with the addition of *Daphnia obtusa* (Alive daphnids), no daphnids (Strains only), pieces of *Daphnia* carapax (Carapax) and gut pieces (Gut). Dark gray shows ED1 strains and light gray shows ED157 strains labeled with fluorescent proteins.

**(iv) Coexistence of ED1 and ED157.** We then conducted an additional experiment where we combined both strains ED1-gfp and ED157-gfp with ED157-DsRed and incubated them either with no animals, live animals, dissected guts, or dissected carapax (Fig. 4D). At days 6 and 8, the abundances of culturable ED1 and ED157 (average of both tag combinations) were not statistically different (Fig. 7, Table 2 and 3), whereas differences in treatments were visible: all treatments were different from each other, except for the one with no *Daphnia* and the one with carapax pieces (Table 2 and 3). Living animals caused faster reduction of E. coli abundances in the surrounding water than the other treatments. E. coli growth with carapax pieces increased in numbers notwithstanding the presence of other bacteria, reaching numbers that were very similar to the treatment containing only the tagged strains. In the presence of gut pieces and naturally associated gut bacteria, the abundances of both strains were reduced much more rapidly, and at the end were similar to the those in the live *Daphnia* treatment (Fig. 7).

**TABLE 2:** Statistical output from the generalized linear model made for the coexistence experiment to evaluate the dependence of E. coli abundance on the treatment (alive, carapax, gut, and strains only), strain (ED1 and ED157), and sampling date (May 24 and 26)^*a*^

Parameter	Chi-square value	df^*b*^	*P* value	Significance^*c*^
Treatment	52.2	3	<0.0001	***
Strain	1.2	1	0.2830	n.s.
Date	4.8	1	0.0284	*

a This is a type 2 analysis of variance table with Wald chi-square tests for predictors.

b df, degrees of freedom.

c ***, *P*  < 0.001; *, *P*  < 0.05; not significant (n.s.), *P*  > 0.05.

**TABLE 3:** Significance of differences in pairwise comparisons between the four treatments as determined by a Tukey’s *post hoc* test^*a*^

Treatment group	Carapax	Gut	Strains only
Alive	***	***	***
Carapax		*	n.s.
Gut	*		***

a ***, *P*  < 0.001; *, *P*  < 0.05; not significant (n.s.), *P*  > 0.05.

## DISCUSSION

In this study, we tackled the question whether *Daphnia* was a host for E. coli in freshwater. One one hand, this bacterium is known to not be competitive in such environments, and to be easily grazed when entering freshwater through fecal pollution (19, 21); however, the evolution (14) and renaturation of freshwater E. coli have been observed (11, 41), showing that at least some incoming E. coli must survive over longer periods in water. In a previous study, we found that lake zooplankton could show remarkable quantities of E. coli-related 16S rRNA gene sequences, and we confirmed this here by quantifying the abundance of *uidA* gene, an unambiguous indicator for E. coli presence (Fig. 1) (39). Indeed, this is not the first time that E. coli has been found to be associated with zooplankton (12). Evidence is accumulating that the freshwater zooplankton microbiota is rather flexible in terms of composition (34, 42–44), meaning that an association with zooplankton might be an interesting potential niche for the short-term survival of FIB. Such a habitat offers protection from protistan grazing and higher nutrient concentrations thanks to filtration feeding from the animal (45). Furthermore, surface attachment is generally considered favorable for the survival of such bacteria compared to a planktonic lifestyle (23, 46). Thus, we tested here whether the association with *Daphnia* allowed E. coli to survive longer in aquatic habitats compared to when the animal was not present. In this study, we found that *Daphnia* can function as a short-term and transitional host for E. coli: as hypothesized, *Daphnia* did reduce E. coli abundances in the surrounding waters, but it was not responsible for the complete removal of E. coli, since many bacterial cells survived gut passages (Fig. 5) and E. coli was still detected after 10 days of coculturing with *Daphnia* (Fig. 5 and 6). In this study, we confirmed that E. coli localizes mainly in the gut of *Daphnia* and that at least part of its population survives the gut passage (37, 38). However, the association with *Daphnia obtusa* did not seem to give a long-term advantage in E. coli survival (Fig. 6 and 7).

Many studies have recently suggested the use of *Daphnia* as a biological control mechanism for E. coli contamination in water: experiments using very large densities of animals and bacteria have shown that the abundance of E. coli was reduced due to grazing by *Daphnia* (37, 38, 47). The addition of *Daphnia* is surely feasible for reducing large abundances of E. coli, but here we showed that E. coli persisted even in the presence of *Daphnia* at low abundances that were more similar to those found in nature. In fact, we also showed that E. coli was still culturable from the gut, even if the bacterium was in very low abundance in the surrounding water. In our environmental survey, we found it to be associated with different zooplankton hosts, especially cladocerans and rotifers (Fig. 1), but it was particularly abundant in a specific sample of zooplankton from Lake Maggiore (Fig. 1). However, also other samples from Lake Maggiore were analyzed in a large study of zooplankton-associated microbes (34), and we did not find any E. coli in these; moreover, the frequency of E. coli in many samples was very low, and the possibility cannot be excluded that some of these were contaminations. This shows that the short-term association can also occur in nature and, consequently, might also spread E. coli which enter the system through superficial contamination due to the animals’ vertical and horizontal migration (31). However, there does not seem to be actual persistence of these bacteria, indicating that the occurrences in the gut are rather stochastic events and that E. coli does not form part of the general *Daphnia* microbiota.

In the experiment where we incubated E. coli with dissected *Daphnia* guts, we observed a similar reduction of bacteria (after 8 days) to that seen with live *Daphnia* (Fig. 7). These data might mean that competition with the gut microbiota was the main reason for reduced E. coli abundance, or that E. coli suffered from digestive enzymes released from the gut. Another interesting finding was that E. coli seemed to profit from the presence of *Daphnia* carapax pieces, which are composed mostly of chitin. Both strains grew better in the presence of carapax despite lacking chitinolytic enzymes in their genomes. It is more likely that the two strains indirectly profited from chitin degradation, since such degradation is usually more efficient when done by multiple species (48) and many bacteria are known to profit from these compounds without being directly involved in the primary degradation (49). The presence of poly-beta-1,6-*N-*acetyl*-*d-glucosamine protein families, which are involved in the synthesis, export, and localization of PGA polymer, shows that these E. coli strains might also be involved in multispecies biofilm formation (50).

We isolated E. coli from *Daphnia* collected from a small pond, albeit with major difficulty; very often, we did not isolate any E. coli. Despite multiple isolation campaigns and the clear presence of E. coli, confirmed by amplification of the *uidA* gene in these pond daphnids, we only isolated 10 strains in total (Fig. 2). This could mean that E. coli associated with *Daphnia* were in a viable but nonculturable state (VBNC), which has been observed in other freshwater environments (51) or when they are exposed to sunlight (52). For Enterococcus faecalis, it was shown that much higher numbers have been detected attached to plankton using culture-independent methods compared to the culturable fraction of these bacteria, and it has been suggested that this attachment in a VBNC state is a survival mode for this species in freshwater (53). A similar situation might also be true for E. coli.

The E. coli strains isolated from *Daphnia* in this study belonged to phylogroups D/E or B1. The analysis of our *Daphnia*-derived E. coli strains themselves did not strongly indicate that these were environmental strains. Touchon and colleagues have shown that freshwater E. coli strains have a reduced genome (14), a typical form of adaptation to oligotrophic environments (16, 18). In an experimental system, Baumgartner and colleagues showed that such genome reduction was quite fast when bacteria were under predation (only a few hundred generations [18, 54]). In the case of our E. coli strains, their genomes were of intermediate size, meaning that they were smaller than those of poultry meat-derived E. coli, but larger than those of freshwater E. coli, which could indicate a certain transition to genome adaptation. The two *Daphnia*-associated E. coli genomes analyzed did not cluster with the freshwater isolates but did cluster with the poultry meat isolates. In fact, the E. coli strains isolated from freshwater might also derive from avian feces (55) and survive associated with zooplankton for a short time. The E. coli genomes also showed some traits that were considered important for adapting to different environments, e.g., the presence of protein families related to the production of capsular polysaccharides (colanic acid), which protect the bacteria from several environmental stress factors (13, 56); and of protein families linked to the type I fimbriae system, which allows bacteria to attach to several eukaryotic cells (57). Whether genetic adaptation of FIB to the environment occurs in zooplankton is an interesting question arising from this study.

Overall, our results showed that the FIB E. coli, when released into the aquatic environment, can form a short-term association with zooplankton, e.g., *Daphnia.* We demonstrated that E. coli does not belong to the core microbiota of *Daphnia*, and it suffers from competition by the natural microbiota of *Daphnia*, but it may resist passage through its gut and benefit from its carapax to survive in water. This association did not prolong their long-term survival in our experiments, but it might provide a niche where these bacteria can encounter other aquatic bacteria, a possible location for horizontal gene transfer. The presence of mobile genetic elements in their genomes, such as plasmids and phage sequences, could suggest lateral gene-transfer events that could play a role in bacterial evolution and speciation (58).

## MATERIALS AND METHODS

### E. coli abundance in zooplankton microbiomes.

Initially, we observed a large number of E. coli/*Shigella* related reads in our Illumina MiSeq data set of zooplankton (*Daphnia* gr. *galeata*/*longispina* and copepod)-associated microbiota obtained from Lake Maggiore (39). To confirm the presence of E. coli, quantitative PCR qPCR assays were conducted using E. coli-specific primers for the *uidA* gene (1-CAATGGTGATGTCAGCGTT and 2-ACACTCTGTCCGGCTTTTG [59]) using the RT-Thermocycler CFX Connect (Bio-Rad). The standard calibration curve for the quantification of *uidA* was carried out as previously described (60) and gene concentration was expressed as gene copy/*Daphnia* or mL of water by dividing the total gene count by the number of animals tested together (20 to 30 animals). The same was done for *Daphnia obtusa* isolated from a small pond in the garden at the Council of Italy Water Research Institute (CNR-IRSA) (35). Twenty individuals were washed in autoclaved MilliQ water, re-collected per triplicate, and introduced in the DNA isolation kit in the Ultra Clean Microbial Kit or the Power Soil DNA Isolation Kit (Qiagen) for DNA extraction. The correct size of all qPCR products was evaluated by electrophoresis (30 min at 80 V, 1% agarose gel). The efficiency of the reaction was 87.5% and the *R*^2^ was 0.99. The limit of quantification (LOQ) was determined (61) to be 45 gene copies/μL.

Furthermore, we checked a large data set of microbial communities associated with zooplankton, taken from many natural freshwater habitats and cultures published elsewhere (34), looking for the presence of E. coli/*Shigella*-affiliated reads. The data set is composed of cladocerans (*Daphnia magna*, *Daphnia obtusa*, *Diaphanosoma brachyurum*, *Simocephalus* sp., and *Mesocyclops leukarti*) and rotifers (*Adineta vaga*, *Keratella serrulata*, *Lecane elsa*, *Lecane inermis*, *Epiphanes senta*, *Keratella quadrata*, and *Polyarthra* sp., see also Fig. 1). Here, we were interested in the maximum potential occurrence of E. coli, thus we counted its presence even if only one replicate showed few E. coli reads (>8 reads in data set).

### E. coli isolation.

Individuals of *Daphnia obtusa* were collected two or three times per week, from May to July and from October to November, from a rainwater-fed pond in the garden of the CNR-IRSA in Verbania (Italy). *D. obtusa* was chosen because, in contrast to *D. galeata/longispina* which can only be found in Lake Maggiore for a short period, *D. obtusa* is always present in the garden ponds and was also shown to contain E. coli. Thirty individuals of *D. obtusa* were washed in autoclaved MilliQ water (Millipore), crushed, and sonicated (3 times, 1 min each cycle with vortexing within cycles) in 1 mL of physiological solution. Serial 10-fold dilutions were performed from 1:10 to 1:10^6^. One mL of each dilution was filtered onto nitrocellulose membrane filters (type GSWP, 25-mm diameter, 0.22-μm pore size, Millipore), and filters were plated onto mFC Agar plates (Biolife) and incubated for 48 h at 37°C.

### Isolate characterization and tagging.

**(i) Identification and genetic characterization of** **E. coli** **isolates.** Aliquots of presumptive E. coli colonies were introduced in 1 mL of physiological solution, centrifuged (5,000 relative centrifugal force [rcf], 4°C for 10 min), boiled for 15 min, frozen for 2 to 4 h, and centrifuged again. DNA from presumptive E. coli colonies and from *Daphnia* was tested for the presence of *uidA* by PCR, using the primers previously mentioned. The conditions for the PCR assays were as follows: 5 μL of Buffer 5×, 0.5 μL deoxynucleoside triphosphate (dNTP; 10 mM), 0.2 μL Taq-polymerase (5 U/μL), 0.25 μL of each primer (100 μM), and water, which was added to arrive to a final volume of 23 μL. PCRs were performed as follows: denaturation for 3 min at 95°C; 35 cycles of 30 s at 95°C, 1 min at 58°C and 1 min at 72°C; and a final extension step of 7 min at 72°C. PCR products were separated by agarose gel electrophoresis (1%) and visualized with GelRed (Midori Green Advance DNA stain). In order to assign specific phylogroups or clades to the 10 E. coli strains isolated, we used the PCR-based method described by Clermont et al. (62). PCR products were separated by agarose gel electrophoresis (1%) and visualized with GelRed (Midori Green Advance DNA stain). The 10 E. coli isolates were further analyzed to obtain an unambiguous DNA fingerprint by enterobacterial repetitive intergenic consensus (ERIC)-PCR, as previously described (63). ERIC-PCR products were separated by electrophoresis for 8 h at 40 V/cm, in 2% agarose Tris borate-EDTA (TBE) gel stained with GelRed (Midori Green Advance DNA stain).

The strains ED1, ED4, ED8, ED157, and ED166 were chosen for multi-locus sequence typing by sequence analysis of the internal fragments of seven housekeeping genes (*adk*, *fumC*, *gyrB*, *icd*, *mdh*, *purA*, *recA*) according to the Achtman scheme (http://enterobase.warwick.ac.uk/species/index/ecoli). The allelic profiles of these genes and the resulting sequence types were determined from the sequence data submitted on the PubMLST database (https://pubmlst.org).

**(ii) Pathogenicity assay.** The hemolytic activity of the strains was evaluated as described by Ghosh et al. (64), with some modifications. Briefly, 4 mL of freshly drawn, heparinized human blood was diluted with 25 mL of phosphate-buffered saline (PBS; pH 7.4). After three washes in 25 mL PBS, the pellet was resuspended in PBS to 20% vol/vol. A 100-μL volume of erythrocyte suspension was added to 100 μL of the bacterial strains. PBS and 0.2% Triton X-100 were used as the negative and positive controls, respectively. After 1 h of incubation at 37°C, each well was centrifuged at 1,200 × *g* for 15 min, then the supernatant was diluted 1:3 in PBS and transferred to a new plate. The optical density at 350 nm (OD_350_) was determined using the Synergy HT microplate reader spectrophotometer (BioTek, Winooski, VT, USA). The hemolysis (%) was determined by the following equation:

$$\left[\left(A\;-\;A_{0}\right)/\left(A_{\text{total}}-\;A_{0}\right)\right]\;\times \;100$$

where *A* is the absorbance of the test well, *A*_0_ the absorbance of the negative control, and *A*_total_ the absorbance of the positive control; the mean value of three replicates was recorded.

To detect biofilm development, the strains were grown in Luria Broth (LB, Merck Life Science) (Oxoid), adjusted to 5 × 10^6^ CFU/mL, and inoculated (100 μL) in 24-well polystyrene plates (VWR International). After 24 h of incubation at 37°C and 24°C, the wells were washed with PBS to eliminate unattached cells, covered with 0.1% (vol/vol) crystal violet (CV) dissolved in H_2_O for 15 min, washed in PBS, and air-dried. The remaining CV was dissolved in 85% ethanol for 15 min at room temperature and 200 μL from each well was transferred to a 24-well plate for spectrophotometric quantification at 570 nm (Multiscan EX Microplate Reader; Thermo Fisher Scientific, Waltham, MA, USA). The strains were classified as strong, moderate, or weak biofilm producers based upon the ODs of the bacterial biofilms (65). All assays were performed in triplicate using independent cultures.

**(iii) Genome sequencing and analysis.** Two strains, namely ED1 and ED157, were chosen for genome sequencing. These two strains were selected because both were affiliated with phylotype D. We decided to further analyze the strains belonging to phylotype D because, compared to others (e.g., B1), this phylotype contains strains which are less commonly recovered from aquatic environments and can originate from a variety of sources, including humans, wild and farm animals, and wastewater (7). We were more interested in these strains because of their respective sequence types: ED1 belonged to a sequence type that contained many bacteria isolated from mammals, including humans, while ED157 belonged to a sequence type which only contained one other E. coli strain that was isolated from water. The strains were grown in LB overnight and DNA extraction was performed using an UltraClean Microbial DNA extraction kit (Qiagen).

Purified DNA was sequenced on a NovaSeq Illumina Platform (IGA Technologies, Padova, Italy), providing a total of 10 and 15 million output reads for ED1 and ED157, respectively. One of these two genomes was already mentioned in a previous article (66). Briefly, reads were first trimmed using Trimmomatic (67) and the genomes were assembled using SPAdes default parameters (68), obtaining totals of 54 and 59 assembled contigs of >1 Kb, respectively.

To verify the phylotypes of E. coli strains ED1 and ED157, we submitted the genome sequences to the website ClermoTyper (69). Antimicrobial resistance genes were identified using ResFinder4.0 (70) and virulence genes were found using the VirulenceFinder 2.0 platform (71). We looked for the presence of elements such as plasmids and phage sequences in the genome sequences, since these can give clues on genome plasticity. Plasmid presence in the genomes was examined using the platform PlasmidFinder (72), and phage genome sequences were recognized using PHASTER (73); these were analyzed since phages play a relevant role in lateral gene transfer (74).

In order to evaluate whether the *Daphnia*-associated isolates were similar to other freshwater E. coli, we compared their genomes to those of other D phylotype strains mentioned by Touchon et al. (14): (i) C4_38 and C2_45, isolated from poultry meat; and (ii) E5895 and E6003, isolated from freshwater (14; Table 1). Strains E5895 and E6003 were, indeed, randomly selected as representative E. coli strains adapted to a freshwater environment, owing to their reduced genomes; the other two strains (C4_38 and C2_45) were randomly selected as representatives of strains from poultry meat, which are known to have the largest average genomes within the E. coli species (74).

Phylogenetic analysis considering the whole genome sequences was performed through the MICROSCOPE platform (http://www.genoscope.cns.fr/agc/microscope [75, 76]). The phylogenetic tree was built using the “genome clustering” MICROSCOPE tool. Genomic similarity was estimated using Mash, with distances correlated with average nucleotide identity (ANI), like *D* ≈ 1–ANI. From all of the pairwise distances of the genome set, a tree was constructed dynamically using the neighbor-joining javascript package, displaying clustering annotations. This clustering was computed from all-pairs distances ≤ 0.06 (≈94% ANI), which correspond to the ANI standard for defining a species group (58). Clustering was computed using Louvain community detection.

To obtain evidence of differences and examine the distribution of protein families across the E. coli genomes indicated in Table 1, we used the “Protein Family Sorter” tool of PATRIC (https://www.patricbrc.org/), setting genus-specific families (PLfams) (77, 78).

**(iv) GFP- and DsRed-tagging of strains.** Competent E. coli ED1 and ED157 cells were prepared in LB medium following the protocol described by Favia et al. (79). Sixty μL of competent cells (∼10^10^ cells · mL^−1^) was mixed with 100 to 200 ng of plasmid DNA, transferred to a cold 0.1-cm-diameter cuvette, and pulsed at 1,700 V in an Electroporator 2510 apparatus (Eppendorf, Milan, Italy). The plasmids used were pHM2-Gfp (79) and pKan(DsRed) (80). Following the pulse, cells were immediately supplemented with 1 mL of LB medium and incubated at 37°C for 1 h. Transformants were selected by plating on LB agar medium supplemented with (i) 100 μg · mL^−1^ kanamycin (KMY), 40 μg · mL^−1^ 5-bromo-4-chloro-3-indolyl-b-d-galactopyranoside (X-Gal), and 0.5 mM isopropyl-b-d-thiogalactopyranoside (IPTG) in the case of plasmid pHM2-Gfp; or with (ii) 100 μg · mL^−1^ KMY in case of plasmid pKan(DsRed). The presence of pHM2-Gfp or pKan(DsRed) plasmids was verified by observing bacterial cells via fluorescence microscopy. Furthermore, the identity of E. coli transformants was confirmed via BOX-PCR amplification (81), comparing their BOX-PCR profiles with those of wild-type ED1 and ED157 strains.

### *Daphnia*-E. coli association experiments.

All laboratory experiments were carried out using *Daphnia obtusa* from the garden of CNR-IRSA. The animals were collected 2 days before the experiment to adapt them to lab conditions. They were washed with artificial lake water medium (inorganic compounds in a previously described composition [82]), fed with a small amount of washed *Kirchneriella* sp., and kept in the dark before experimental use. The animals were washed again in ALW before each experiment and the experiments were conducted in the same medium unless otherwise specified. The E. coli strains ED1 and ED157 tagged with fluorescent proteins which were used in the experiments were grown overnight at 37°C in liquid LB containing 100 μg · mL^−1^ KMY to maintain the plasmids. The strains were centrifuged and washed twice with ALW before inoculation in experimental treatments on the following day. All experiments were carried out at room temperature in the dark. Fig. 4 illustrates the approaches taken for the presented laboratory experiments. All figures of the experiments were drawn in *R* (83), using the packages *ggplot*2 (84), *reshape*2 (85), and *cowplot* (86).

**(i) Localization of** **E. coli** **on *Daphnia.*** We verified where E. coli attached to *Daphnia* by incubating E. coli with live *Daphnia* for 4 h. Animals were then dissected, DNA was extracted using the UltraClean Microbial DNA kit (Qiagen), and DNA from different body parts was subjected to qPCR amplification of the *gfp* gene to verify the presence of and quantify E. coli. qPCR assays were carried out in a volume of 20 μL containing 2 μL of DNA, 0.5 μM of each primer (1-GAAGATGGAAGCGTTCAA and 2-AGGTAATGGTTGTCTGGTA [87]), 10 μL of SsoAdvanced Universal SYBR Green Supermix (Bio-Rad), and filtered and autoclaved MilliQ water (Millipore) to the final volume. The qPCR program was 95°C for 2 min, 35 cycles of 95°C for 15 s, 54°C for 30 s, and 72°C for 15 s. A melting curve was performed from 60°C to 95°C in increments of 0.5°C/5 s. The correct size of all qPCR amplicons was evaluated by an electrophoresis run (carried out as described above for *uidA*). The standard curve for the *gfp* gene quantification was carried out by dilution of the purified and quantified amplicon, as performed for the *uidA* gene and previously described in Sabatino et al. (60). The reaction efficiency was 91% and the *R*^2^ was 0.99. The LOQ (determined as described above for the *uidA* gene) was 9.85 gene copies/μL. The concentration of the *gfp* gene was expressed as no. gene copies/*Daphnia*. We also verified differences in attachment to gut and carapax by epifluorescence microscopy.

**(ii) Release of** **E. coli** **after gut passage.** We then tested whether E. coli was a food source for *Daphnia*, whether *Daphnia* functioned as a refuge for E. coli, and whether E. coli simply passed through the gut or could be transferred from other body parts. First, we incubated ED1-gfp and ED157-gfp separately, with and without *Daphnia*, in 50 mL ALW for 2 h in the dark at room temperature. Next, we transferred 50 μL of water, either alone or with a single *Daphnia* (+D treatment or control) and, after another 2 h of incubation, compared the amount of ED1-gfp transferred into the surrounding water. ED1-gfp was counted on a flow cytometer as green fluorescent events (BD C6, Accuri). Differences between treatments were evaluated using a linear model on log-transformed count data for the response variable, conducted in R (Table 3).

**(iii) Persistence of** **E. coli** **with *Daphnia.*** The chemostat was a continuous culturing system with three medium tanks containing ALW attached to six vessels containing 700 mL of medium. A non-axenic *Kirchneriella* culture was added to both the medium and vessels at a density of around 20,000 cells per mL (day T –5). The system was kept in the dark. After 3 days (day T –2), the chemostat pumps were switched on with a daily water replacement rate of 10%. After 1 day (T –1), ED1-gfp was added to the vessel at a concentration of 0.5 × 10^6^ cells mL^−1^, as well as algae, to maintain around 20,000 cells mL^−1^. This experiment was conducted with strain ED1 because, according to its sequence type, it is a more relevant potential contaminant of mammalian origin in freshwater. After another day (T0), *Daphnia obtusa* was added to the vessels, which were randomly assigned with a quantity of animals in a gradient with the following numbers of animals per vessel: 0, 2, 6, 8, 10, and 15. Samples of 40 mL each were taken every 2 to 3 days over the outflow of the chemostat vessel, and *Kirchneriella* solution was always added after sampling to maintain food for *Daphnia*. These samples were used for CFU counts for ED1-gfp and for microscopy counts for both algae and ED1. For phytoplankton counts, 10 mL solution was filtered on 0.45-μm pore polycarbonate filters and at least 10 fields and 500 cells were counted at a magnification of 80,000× on an epifluorescence microscope (Zeiss). To determine CFU on days 0 and 2, 5-μL spots of a gradual dilution between 1 and 10^−4^ were spotted on LB+KMY plates, grown for 24 h at 37°C, and counted. The presence of *gfp* and thus the univocal identification of ED1-gfp was determined by placing the plate on a transilluminator (UV light) and observing the green fluorescence of the colonies. Due to the strong reduction of E. coli CFU, on days 5 and 7 CFU were counted by filtration of 1 mL of undiluted and 1:10 diluted sample, and on day 9 by filtration of 1 mL of undiluted and 10 mL of undiluted sample, on a 0.2-μm pore nitrocellulose membrane filter placed on the plates, and colonies were counted as described above. All spots and filters were performed in triplicate per sample. By T12 *Daphnia* numbers were strongly reduced (see Table S1), thus the experiment was considered finished. Individuals of *Daphnia* were extracted from the vessels, washed 3 times with sterile ALW, and then dissected; the different body parts were placed in 200 μL LB+KMY on a black multiwell-plate in a plate reader (GlowMax, Promega). E. coli growth was detected by monitoring fluorescence over 48 h every 30 min. The total number of dissected *Daphnia* was 12 adults (3 from vessel 6, 4 from vessel 5, 4 from vessel 3 and 1 from vessel 1) and 9 juveniles (1 from vessel 6, 4 from vessel 5, 2 from vessel 3); 7 negative controls were included. We checked the influence of the original gradient of *Daphnia* abundance on the CFU of E. coli by a generalized linear model assuming a negative binomial distribution of the data. The model was evaluated using *check_model* from the *performance* package (88) and the output was depicted as a type II analysis of variance table using the *car* package (89).

**(iv) Coexistence experiment.** In order to test whether ED1 and ED157 reacted similarly to the presence of *Daphnia* and its associated bacteria, we conducted a batch experiment in which we incubated both strains together with no *Daphnia*, live *Daphnia*, and dissected *Daphnia*, for which we made one treatment containing *Daphnia* guts and one containing *Daphnia* carapax and filtration apparatus and their associated bacteria. Each replicate was amended with either 3 live *Daphnia* or dissection pieces from 10 *Daphnia* in 2 mL 1:100 diluted LB with ALW and in triplicate. Moreover, each treatment was conducted twice once using ED1-gfp + ED157-DsRed and once using ED1-DsRed and ED157-gfp to account for potential differences in fitness reduction due to the two different fluorescence markers (total treatment: *n* = 2 stainings × 3 replicates × 4 treatments = 24). We used 5 × 10^7^ cells · mL^−1^ for each strain; higher concentrations of cells and a more nutrient-rich medium were chosen in order to have generally higher numbers of E. coli that were easier to track on plate counts. In fact, in both cases, ED1-DsRed had a fitness advantage; thus, the numbers presented here are averages between the CFU counted for -gfp and -DsRed of the same strain in the same treatment. CFU were counted over 10 days, beginning on T4, by spotting of 5 μL diluted up to 10^−8^ in triplicate, and green and red colonies were counted on a trans-illuminator (UV). The experiment was stopped after 10 days due to major mortality of *Daphnia* in the live treatment (>90%), and data for the first 8 days were plotted. To evaluate long-term differences between treatments, data from March 24 and 26 were used (6 and 8 days). A generalized linear model assuming a negative binomial distribution of the data was made to evaluate the effects of the treatment and the date on the abundance of both E. coli ED1 and ED157 (*glm.nb* in R with the model: CFU ∼ treatment [4 levels: alive, carapax, gut, no *Daphnia*] + strain [2 levels: ED1 or ED157] + date [2 levels: March 24 and 26]). Model check and model output were performed as for the analysis of persistence of E. coli with *Daphnia*; in addition, pairwise differences between treatments were evaluated with a *post hoc* test using *emmeans* from the homonymous package (90).

### Data and code accessibility.

All scripts and raw data have been deposited at https://github.com/EsterME/E_coli_Daphnia. The ED1 genome was deposited into the NCBI GenBank database under the accession no. JAAWVB00000000 (66). The ED157 Genome Shotgun project has been deposited in DDBJ/ENA/GenBank under the accession no. JABEXY000000000. The version described in this paper is version JABEXY010000000.

## References

[1] Korajkic A, Wanjugi P, Brooks L, Cao Y, Harwood Valerie J. 2019. Persistence and decay of fecal microbiota in aquatic habitats. Microbiol Mol Biol Rev 83:e00005-19. 10.1128/MMBR.00005-19. 31578217 PMC7405076

[2] Martinson JN, Walk ST. 2020. *Escherichia coli* residency in the gut of healthy human adults. EcoSal Plus 9:10.1128/ecosalplus.ESP-0003-2020. 10.1128/ecosalplus.ESP-0003-2020. PMC7523338 32978935

[3] Espinosa-Urgel M, Kolter R. 1998. *Escherichia coli* genes expressed preferentially in an aquatic environment. Mol Microbiol 28:325–332. 10.1046/j.1365-2958.1998.00796.x. 9622357

[4] Jang J, Hur HG, Sadowsky MJ, Byappanahalli M, Yan T, Ishii S. 2017. Environmental *Escherichia coli*: ecology and public health implications: a review. J Appl Microbiol 123:570–581. 10.1111/jam.13468. 28383815

[5] Vignaroli C, Luna G, Pasquaroli S, Di Cesare A, Petruzzella R, Paroncini P, Biavasco F. 2013. Epidemic *Escherichia coli* ST131 and *Enterococcus faecium* ST17 in coastal marine sediments from an Italian beach. Environ Sci Technol 47:13772–13780. 10.1021/es4019139. 24195439

[6] Jørgensen SB, Søraas AV, Arnesen LS, Leegaard TM, Sundsfjord A, Jenum PA. 2017. A comparison of extended spectrum β-lactamase producing *Escherichia coli* from clinical, recreational water and wastewater samples associated in time and location. PLoS One 12:e0186576. 10.1371/journal.pone.0186576. 29040337 PMC5645111

[7] Vignaroli C, Luna GM, Rinaldi C, Di Cesare A, Danovaro R, Biavasco F. 2012. New sequence types and multidrug resistance among pathogenic *Escherichia coli* isolates from coastal marine sediments. Appl Environ Microbiol 78:3916–3922. 10.1128/AEM.07820-11. 22447595 PMC3346399

[8] Baniga Z, Hounmanou YMG, Kudirkiene E, Kusiluka LJM, Mdegela RH, Dalsgaard A. 2020. Genome-based analysis of extended-spectrum β-lactamase-producing *Escherichia coli* in the aquatic environment and Nile perch (*Lates niloticus*) of Lake Victoria, Tanzania. Front Microbiol 11:108. 10.3389/fmicb.2020.00108. 32153519 PMC7046833

[9] Fernandes MR, Sellera FP, Moura Q, Esposito F, Sabino CP, Lincopan N. 2020. Identification and genomic features of halotolerant extended-spectrum-β-lactamase (CTX-M)-producing *Escherichia coli* in urban-impacted coastal waters, Southeast Brazil. Mar Pollut Bull 150:110689. 10.1016/j.marpolbul.2019.110689. 31733900

[10] Power ML, Littlefield-Wyer J, Gordon DM, Veal DA, Slade MB. 2005. Phenotypic and genotypic characterization of encapsulated *Escherichia coli* isolated from blooms in two Australian lakes. Environ Microbiol 7:631–640. 10.1111/j.1462-2920.2005.00729.x. 15819845

[11] Ishii S, Ksoll WB, Hicks RE, Sadowsky MJ. 2006. Presence and growth of naturalized *Escherichia coli* in temperate soils from Lake Superior watersheds. Appl Environ Microbiol 72:612–621. 10.1128/AEM.72.1.612-621.2006. 16391098 PMC1352292

[12] Ishii S, Yan T, Shively DA, Byappanahalli MN, Whitman RL, Sadowsky MJ. 2006. *Cladophora* (Chlorophyta) spp. harbor human bacterial pathogens in nearshore water of Lake Michigan. Appl Environ Microbiol 72:4545–4553. 10.1128/AEM.00131-06. 16820442 PMC1489363

[13] Walk ST, Alm EW, Calhoun LM, Mladonicky JM, Whittam TS. 2007. Genetic diversity and population structure of Escherichia coli isolated from freshwater beaches. Environ Microbiol 9:2274–2288. 10.1111/j.1462-2920.2007.01341.x. 17686024

[14] Touchon M, Perrin A, de Sousa JAM, Vangchhia B, Burn S, O'Brien CL, Denamur E, Gordon D, Rocha EP. 2020. Phylogenetic background and habitat drive the genetic diversification of Escherichia coli. PLoS Genet 16:e1008866. 10.1371/journal.pgen.1008866. 32530914 PMC7314097

[15] Grote J, Thrash JC, Huggett MJ, Landry ZC, Carini P, Giovannoni SJ, Rappé MS. 2012. Streamlining and core genome conservation among highly divergent members of the SAR11 clade. mBio 3:e00252-12. 10.1128/mBio.00252-12. 22991429 PMC3448164

[16] Salcher MM, Schaefle D, Kaspar M, Neuenschwander SM, Ghai R. 2019. Evolution in action: habitat transition from sediment to the pelagial leads to genome streamlining in Methylophilaceae. ISME J 13:2764–2777. 10.1038/s41396-019-0471-3. 31292537 PMC6794327

[17] Lee M-C, Marx CJ. 2012. Repeated, selection-driven genome reduction of accessory genes in experimental populations. PLoS Genet 8:e1002651. 10.1371/journal.pgen.1002651. 22589730 PMC3349727

[18] Baumgartner M, Roffler S, Wicker T, Pernthaler J. 2017. Letting go: bacterial genome reduction solves the dilemma of adapting to predation mortality in a substrate-restricted environment. ISME J 11:2258–2266. 10.1038/ismej.2017.87. 28585936 PMC5607369

[19] González JM, Iriberri J, Egea L, Barcina I. 1992. Characterization of culturability, protistan grazing, and death of enteric bacteria in aquatic ecosystems. Appl Environ Microbiol 58:998–1004. 10.1128/aem.58.3.998-1004.1992. 1575503 PMC195368

[20] Wanjugi P, Harwood VJ. 2013. The influence of predation and competition on the survival of commensal and pathogenic fecal bacteria in aquatic habitats. Environ Microbiol 15:517–526. 10.1111/j.1462-2920.2012.02877.x. 23013262

[21] Eckert EM, Quero GM, Di Cesare A, Manfredini G, Mapelli F, Borin S, Fontaneto D, Luna GM, Corno G. 2019. Antibiotic disturbance affects aquatic microbial community composition and food web interactions but not community resilience. Mol Ecol 28:1170–1182. 10.1111/mec.15033. 30697889

[22] Roslev P, Bjergbæk LA, Hesselsoe M. 2004. Effect of oxygen on survival of faecal pollution indicators in drinking water. J Applied Microbiology 96:938–945. 10.1111/j.1365-2672.2004.02209.x. 15078509

[23] Costerton JW, Stewart PS, Greenberg EP. 1999. Bacterial biofilms: a common cause of persistent infections. Science 284:1318–1322. 10.1126/science.284.5418.1318. 10334980

[24] Hall-Stoodley L, Stoodley P. 2005. Biofilm formation and dispersal and the transmission of human pathogens. Trends Microbiol 13:7–10. 10.1016/j.tim.2004.11.004. 15639625

[25] Eckert EM, Di Cesare A, Coci M, Corno G. 2018. Persistence of antibiotic resistance genes in large subalpine lakes: the role of anthropogenic pollution and ecological interactions. Hydrobiologia 824:93–108. 10.1007/s10750-017-3480-0.

[26] Haller L, Poté J, Loizeau J-L, Wildi W. 2009. Distribution and survival of faecal indicator bacteria in the sediments of the Bay of Vidy, Lake Geneva, Switzerland. Ecological Indicators 9:540–547. 10.1016/j.ecolind.2008.08.001.

[27] John P, Haller L, Kottelat R, Sastre V, Arpagaus P, Wildi W. 2009. Persistence and growth of faecal culturable bacterial indicators in water column and sediments of Vidy Bay, Lake Geneva, Switzerland. J Environmental Sciences 21:62–69. 10.1016/S1001-0742(09)60012-7. 19402401

[28] Quero GM, Fasolato L, Vignaroli C, Luna GM. 2015. Understanding the association of *Escherichia coli* with diverse macroalgae in the lagoon of Venice. Sci Rep 5:10969. 10.1038/srep10969. 26043415 PMC4455311

[29] Declerck P, Behets J, van Hoef V, Ollevier F. 2007. Detection of *Legionella* spp. and some of their amoeba hosts in floating biofilms from anthropogenic and natural aquatic environments. Water Res 41:3159–3167. 10.1016/j.watres.2007.04.011. 17544473

[30] Abgottspon H, Nüesch-Inderbinen MT, Zurfluh K, Althaus D, Hächler H, Stephan R. 2014. Enterobacteriaceae with extended-spectrum- and pAmpC-type ß-lactamase-encoding genes isolated from freshwater fish from two lakes in Switzerland. Antimicrob Agents Chemother 58:2482–2484. 10.1128/AAC.02689-13. 24449774 PMC4023715

[31] Grossart HP, Dziallas C, Leunert F, Tang KW. 2010. Bacteria dispersal by hitchhiking on zooplankton. Proc Natl Acad Sci USA 107:11959–11964. 10.1073/pnas.1000668107. 20547852 PMC2900670

[32] Hammer TJ, Sanders JG, Fierer N. 2019. Not all animals need a microbiome. FEMS Microbiol Lett 366:fnz117. 10.1093/femsle/fnz117. 31132110

[33] Callens M, De Meester L, Muylaert K, Mukherjee S, Decaestecker E. 2020. The bacterioplankton community composition and a host genotype dependent occurrence of taxa shape the *Daphnia magna* gut bacterial community. FEMS Microbiol Ecol 96:fiaa128. 10.1093/femsec/fiaa128. 32573725 PMC7360484

[34] Eckert EM, Anicic N, Fontaneto D. 2021. Freshwater zooplankton microbiome composition is highly flexible and strongly influenced by the environment. Mol Ecol 30:1545–1558. 10.1111/mec.15815. 33484584

[35] Eckert EM, Di Cesare A, Stenzel B, Fontaneto D, Corno G. 2016. *Daphnia* as a refuge for an antibiotic resistance gene in an experimental freshwater community. Sci Total Environ 571:77–81. 10.1016/j.scitotenv.2016.07.141. 27459256

[36] Olanrewaju TO, McCarron M, Dooley JS, Arnscheidt J. 2019. Transfer of antibiotic resistance genes between *Enterococcus faecalis* strains in filter feeding zooplankton *Daphnia magna* and *Daphnia pulex*. Sci Total Environ 659:1168–1175. 10.1016/j.scitotenv.2018.12.314. 31096330

[37] Burnet JB, Faraj T, Cauchie HM, Joaquim-Justo C, Servais P, Prévost M, Dorner SM. 2017. How does the cladoceran *Daphnia pulex* affect the fate of *Escherichia coli* in water? PLoS One 12:e0171705. 10.1371/journal.pone.0171705. 28178322 PMC5298254

[38] Ismail NS, Blokker BM, Feeney TR, Kohn RH, Liu J, Nelson VE, Ollive MC, Price SBL, Underdah EJ. 2019. Impact of metazooplankton filter feeding on Escherichia coli under variable environmental conditions. Appl Environ Microbiol 85:e02006-19. 10.1128/AEM.02006-19. 31562176 PMC6856336

[39] Eckert EM, Amalfitano S, Di Cesare A, Manzari C, Corno G, Fontaneto D. 2020. Different substrates within a lake harbour connected but specialised microbial communities. Hydrobiologia 847:1689–1704. 10.1007/s10750-019-04068-1.

[40] Koskiniemi S, Lamoureux JG, Nikolakakis KC, Kint de Roodenbeke C, Kaplan MD, Low DA, Hayes CS. 2013. Rhs proteins from diverse bacteria mediate intercellular competition. Proc Natl Acad Sci USA 110:7032–7037. 10.1073/pnas.1300627110. 23572593 PMC3637788

[41] Ishii S, Sadowsky MJ. 2008. *Escherichia coli* in the environment: implications for water quality and human health. Microbes Environ 23:101–108. 10.1264/jsme2.23.101. 21558695

[42] Callens M, Macke E, Muylaert K, Bossier P, Lievens B, Waud M, Decaestecker E. 2016. Food availability affects the strength of mutualistic host-microbiota interactions in *Daphnia magna*. ISME J 10:911–920. 10.1038/ismej.2015.166. 26405832 PMC4796930

[43] Macke E, Callens M, Massol F, Vanoverberghe I, De Meester L, Decaestecker E. 2020. Diet and genotype of an aquatic invertebrate affect the composition of free-living microbial communities. Front Microbiol 11:380. 10.3389/fmicb.2020.00380. 32256467 PMC7090131

[44] Pfenning-Butterworth A, Cooper RO, Cressler CE. 2022. Daily feeding rhythm linked to microbiome composition in two zooplankton species. PLoS One 17(2): e0263538. 10.1371/journal.pone.0263538. 35113950 PMC8812976

[45] Eckert EM, Pernthaler J. 2014. Bacterial epibionts of *Daphnia*: a potential route for the transfer of dissolved organic carbon in freshwater food webs. ISME J 8:1808–1819. 10.1038/ismej.2014.39. 24694716 PMC4139729

[46] Allen HK, Donato J, Wang HH, Cloud-Hansen KA, Davies J, Handelsman J. 2010. Call of the wild: antibiotic resistance genes in natural environments. Nat Rev Microbiol 8:251–259. 10.1038/nrmicro2312. 20190823

[47] Nørgaard LS, Roslev P. 2016. Effects of ammonia and density on filtering of commensal and pathogenic *Escherichia coli* by the cladoceran *Daphnia magna*. Bull Environ Contam Toxicol 97:848–854. 10.1007/s00128-016-1963-8. 27817111

[48] Corno G, Salka I, Pohlmann K, Hall AR, Grossart H-P. 2015. Interspecific interactions drive chitin and cellulose degradation by aquatic microorganisms. Aquatic Microb Ecol 76:27–37. 10.3354/ame01765.

[49] Beier S, Bertilsson S. 2013. Bacterial chitin degradation: mechanisms and ecophysiological strategies. Front Microbiol 4:149. 10.3389/fmicb.2013.00149. 23785358 PMC3682446

[50] Kang J, Li Q, Liu L, Jin W, Wang J, Sun Y. 2018. The specific effect of gallic acid on *Escherichia coli* biofilm formation by regulating *pgaABCD* genes expression. Appl Microbiol Biotechnol 102:1837–1846. 10.1007/s00253-017-8709-3. 29327068

[51] Liu Y, Gilchrist A, Zhang J, Li X-F. 2008. Detection of viable but nonculturable *Escherichia coli* O157:H7 bacteria in drinking water and river water. Appl Environ Microbiol 74:1502–1507. 10.1128/AEM.02125-07. 18203853 PMC2258616

[52] Pommepuy M, Butin M, Derrien A, Gourmelon M, Colwell RR, Cormier M. 1996. Retention of enteropathogenicity by viable but nonculturable *Escherichia coli* exposed to seawater and sunlight. Appl Environ Microbiol 62:4621–4626. 10.1128/aem.62.12.4621-4626.1996. 8953732 PMC168287

[53] Signoretto C, Burlacchini G, Lleò MM, Pruzzo C, Zampini M, Pane L, Franzini G, Canepari P. 2004. Adhesion of *Enterococcus faecalis* in the nonculturable state to plankton is the main mechanism responsible for persistence of this bacterium in both lake and seawater. Appl Environ Microbiol 70:6892–6896. 10.1128/AEM.70.11.6892-6896.2004. 15528559 PMC525140

[54] Baumgartner M, Neu TR, Blom JF, Pernthaler J. 2016. Protistan predation interferes with bacterial long-term adaptation to substrate restriction by selecting for defence morphotypes. J Evol Biol 29:2297–2310. 10.1111/jeb.12957. 27488245

[55] Meerburg BG, Koene MGJ, Kleijn D. 2011. *Escherichia coli* concentrations in feces of geese, coots, and gulls residing on recreational water in The Netherlands. Vector Borne Zoonotic Dis 11:601–603. 10.1089/vbz.2010.0218. 21548761

[56] Azurmendi HF, Veeramachineni V, Freese S, Lichaa F, Freedberg DI, Vann WF. 2020. Chemical structure and genetic organization of the *E. coli* O6: K15 capsular polysaccharide. Sci Rep 10:1–12. 10.1038/s41598-020-69476-z. 32724125 PMC7387560

[57] Gally DL, Bogan JA, Eisenstein BI, Blomfield IC. 1993. Environmental regulation of the fim switch controlling type 1 fimbrial phase variation in *Escherichia coli* K-12: effects of temperature and media. J Bacteriol 175:6186–6193. 10.1128/jb.175.19.6186-6193.1993. 8104927 PMC206713

[58] Konstantinidis KT, Tiedje JM. 2005. Genomic insights that advance the species definition for prokaryotes. Proc Natl Acad Sci USA 102:2567–2572. 10.1073/pnas.0409727102. 15701695 PMC549018

[59] Srinivasan S, Aslan A, Xagoraraki I, Alocilja E, Rose JB. 2011. *Escherichia coli*, enterococci, and *Bacteroides thetaiotaomicron* qPCR signals through wastewater and septage treatment. Water Res 45:2561–2572. 10.1016/j.watres.2011.02.010. 21420709

[60] Sabatino R, Di Cesare A, Pasquaroli S, Vignaroli C, Citterio B, Amiri M, Rossi L, Magnani M, Mauro A, Biavasco F. 2015. Adherence and intracellular survival within human macrophages of *Enterococcus faecalis* isolates from coastal marine sediment. Microbes Infect 17:660–664. 10.1016/j.micinf.2015.06.001. 26079735

[61] Bustin SA, Benes V, Garson JA, Hellemans J, Huggett J, Kubista M, Mueller R, Nolan T, Pfaffl MW, Shipley GL, Vandesompele J, Wittwer CT. 2009. The MIQE guidelines: minimum information for publication of quantitative real-time PCR experiments. Clin Chem 55:611–622. 10.1373/clinchem.2008.112797. 19246619

[62] Clermont O, Christenson JK, Denamur E, Gordon DM. 2013. The Clermont *Escherichia coli* phylo-typing method revisited: improvement of specificity and detection of new phylo-groups. Environ Microbiol Rep 5:58–65. 10.1111/1758-2229.12019. 23757131

[63] Versalovic J, Koeuth T, Lupski R. 1991. Distribution of repetitive DNA sequences in eubacteria and application to fingerprinting of bacterial enomes. Nucleic Acids Res 19:6823–6831. 10.1093/nar/19.24.6823. 1762913 PMC329316

[64] Ghosh C, Manjunath GB, Akkapeddi P, Yarlagadda V, Hoque J, Uppu DSSM, Konai MM, Haldar J. 2014. Small molecular antibacterial peptoid mimics: the simpler the better! J Med Chem 57:1428–1436. 10.1021/jm401680a. 24479371

[65] Stepanović S, Vuković D, Hola V, Di Bonaventura G, Djukić S, Cirković I, Ruzicka F. 2007. Quantification of biofilm in microtiter plates: overview of testing conditions and practical recommendations for assessment of biofilm production by staphylococci. APMIS 115:891–899. 10.1111/j.1600-0463.2007.apm_630.x. 17696944

[66] Riva F, Riva V, Eckert EM, Colinas N, Di Cesare A, Borin S, Mapelli F, Crotti E. 2020. An environmental *Escherichia coli* strain is naturally competent to acquire exogenous DNA. Front Microbiol 11:574301. 10.3389/fmicb.2020.574301. 33013812 PMC7494812

[67] Bolger AM, Lohse M, Usadel B. 2014. Trimmomatic: a flexible trimmer for Illumina sequence data. Bioinformatics 30:2114–2120. 10.1093/bioinformatics/btu170. 24695404 PMC4103590

[68] Prjibelski A, Antipov D, Meleshko D, Lapidus A, Korobeynikov A. 2020. Using SPAdes *de novo* assembler. Curr Protoc Bioinformatics 70:e102. 10.1002/cpbi.102. 32559359

[69] Beghain J, Bridier-Nahmias A, Le Nagard H, Denamur E, Clermont O. 2018. ClermonTyping: an easy-to-use and accurate in silico method for *Escherichia* genus strain phylotyping. Microb Genom 4:e000192. 10.1099/mgen.0.000192. PMC6113867 29916797

[70] Bortolaia V, Kaas RS, Ruppe E, Roberts MC, Schwarz S, Cattoir V, Philippon A, Allesoe RL, Rebelo AR, Florensa AF. 2020. ResFinder 4.0 for predictions of phenotypes from genotypes. J Antimicrob Chemother 75:3491–3500. 10.1093/jac/dkaa345. 32780112 PMC7662176

[71] Joensen KG, Scheutz F, Lund O, Hasman H, Kaas RS, Nielsen EM, Aarestrup FM. 2014. Real-time whole-genome sequencing for routine typing, surveillance, and outbreak detection of verotoxigenic *Escherichia coli*. J Clin Microbiol 52:1501–1510. 10.1128/JCM.03617-13. 24574290 PMC3993690

[72] Carattoli A, Zankari E, García-Fernández A, Voldby Larsen M, Lund O, Villa L, Møller Aarestrup F, Hasman H. 2014. *In silico* detection and typing of plasmids using PlasmidFinder and plasmid multilocus sequence typing. Antimicrob Agents Chemother 58:3895–3903. 10.1128/AAC.02412-14. 24777092 PMC4068535

[73] Arndt D, Grant JR, Marcu A, Sajed T, Pon A, Liang Y, Wishart DS. 2016. PHASTER: a better, faster version of the PHAST phage search tool. Nucleic Acids Res 44:W16–W21. 10.1093/nar/gkw387. 27141966 PMC4987931

[74] Canchaya C, Fournous G, Chibani-Chennoufi S, Dillmann ML, Brüssow H. 2003. Phage as agents of lateral gene transfer. Curr Opin Microbiol 6:417–424. 10.1016/s1369-5274(03)00086-9. 12941415

[75] Vallenet D, Engelen S, Mornico D, Cruveiller S, Fleury L, Lajus A, Rouy Z, Roche D, Salvignol G, Scarpelli C, Médigue C. 2009. MicroScope: a platform for microbial genome annotation and comparative genomics. Database (Oxford) 2009:bap021. 10.1093/database/bap021. 20157493 PMC2790312

[76] Blondel VD, Guillaume J-L, Lambiotte R, Lefebvre E. 2008. Fast unfolding of communities in large networks. J Statistical Mechanics 2008:P10008. 10.1088/1742-5468/2008/10/P10008.

[77] Davis JJ, Gerdes S, Olsen GJ, Olson R, Pusch GD, Shukla M, Vonstein V, Wattam AR, Yoo H. 2016. PATtyFams: protein families for the microbial genomes in the PATRIC database. Front Microbiol 7:118. 10.3389/fmicb.2016.00118. 26903996 PMC4744870

[78] Davis JJ, Wattam AR, Aziz RK, Brettin T, Butler R, Butler RM, Chlenski P, Conrad N, Dickerman A, Dietrich EM, Gabbard JL, Gerdes S, Guard A, Kenyon RW, Machi D, Mao C, Murphy-Olson D, Nguyen M, Nordberg EK, Olsen GJ, Olson RD, Overbeek JC, Overbeek R, Parrello B, Pusch GD, Shukla M, Thomas C, VanOeffelen M, Vonstein V, Warren AS, Xia F, Xie D, Yoo H, Stevens R. 2020. The PATRIC Bioinformatics Resource Center: expanding data and analysis capabilities. Nucleic Acids Res 48:D606–D612. 10.1093/nar/gkz943. 31667520 PMC7145515

[79] Favia G, Ricci I, Damiani C, Raddadi N, Crotti E, Marzorati M, Rizzi A, Urso R, Brusetti L, Borin S, Mora D, Scuppa P, Pasqualini L, Clementi E, Genchi M, Corona S, Negri I, Grandi G, Alma A, Kramer L, Esposito F, Bandi C, Sacchi L, Daffonchio D. 2007. Bacteria of the genus *Asaia* stably associate with *Anopheles stephensi*, an Asian malarial mosquito vector. Proc Natl Acad Sci USA 104:9047–9051. 10.1073/pnas.0610451104. 17502606 PMC1885625

[80] Crotti E, Damiani C, Pajoro M, Gonella E, Rizzi A, Ricci I, Negri I, Scuppa P, Rossi P, Ballarini P, Raddadi N, Marzorati M, Sacchi L, Clementi E, Genchi M, Mandrioli M, Bandi C, Favia G, Alma A, Daffonchio D. 2009. *Asaia*, a versatile acetic acid bacterial symbiont, capable of cross‐colonizing insects of phylogenetically distant genera and orders. Environ Microbiol 11:3252–3264. 10.1111/j.1462-2920.2009.02048.x. 19735280

[81] Urzì C, Brusetti L, Salamone P, Sorlini C, Stackebrandt E, Daffonchio D. 2001. Biodiversity of Geodermatophilaceae isolated from altered stones and monuments in the Mediterranean basin. Environ Microbiol 3:471–479. 10.1046/j.1462-2920.2001.00217.x. 11553237

[82] Zotina T, Köster O, Jüttner F. 2003. Photoheterotrophy and light‐dependent uptake of organic and organic nitrogenous compounds by *Planktothrix rubescens* under low irradiance. Freshwater Biology 48:1859–1872. 10.1046/j.1365-2427.2003.01134.x.

[83] RCore Team. 2013. R: A language and environment for statistical computing. Vienna, Austria.

[84] Wickham H. 2009. ggplot2: elegant graphics for data analysis. Springer Science & Business Media, Berlin, Germany.

[85] Wickham H. 2020. reshape2: flexibly reshape data: a reboot of the reshape package. R package version 1.4.4. Available from https://github.com/hadley/reshape.

[86] Wilke CO. 2020 cowplot: streamlined plot theme and plot annotations for 'ggplot2'. Available from https://wilkelab.org/cowplot/.

[87] Hale L, Luth M, Crowley D. 2015. Biochar characteristics relate to its utility as an alternative soil inoculum carrier to peat and vermiculite. Soil Biol Biochem 81:228–235. 10.1016/j.soilbio.2014.11.023.

[88] Lüdecke D, Makowski D, Waggoner P. 2019. performance: assessment of regression models performance. R package version 04.2. Available from https://rdrr.io/cran/performance/.

[89] Fox J, Weisberg S, Adler D, Bates D, Baud-Bovy G, Ellison S, Firth D, Friendly M, Gorjanc G, Graves S. 2012. Package ‘car’. R Foundation for Statistical Computing, Vienna, Austria.

[90] Lenth RV. 2020. emmeans: estimated marginal means, aka least-squares means. R package version 1.5.3. Available from https://github.com/rvlenth/emmeans.

