# Supplementary material for: Zooplankton as a Transitional Host for Escherichia coli in Freshwater
Source: Appl Environ Microbiol. 2022 Apr 13;88(9):e02522-21. doi: 10.1128/aem.02522-21 (PMC9088391; doi:10.1128/aem.02522-21)
Supplement: Supplemental file 1 — Tables S1 to S4. Download aem.02522-21-s0001.pdf, PDF file, 0.6 MB [file aem.02522-21-s0001.pdf]

Supplementary files

Supplementary table 1 Numbers of surviving *Daphnia* in the chemostat vessels over time.

Vessel	day 1	day 7	day 13
V1	4	2	1
V2	2	0	0
V3	10	6	4
V4	0	0	0
V5	8	4	3
V6	15	13	3

Supplementary table 2 Details of genes and sequence types found in MLST screening of *E. coli* strains isolated from *Daphnia obtusa*.

	adk	*fumC*	*gyrB*	*icd*	*mdh*	*purA*	*recA*	ST
**ED1**	4	26	2	25	5	5	19	ST38 (ST38 Cplx)
**ED4**	4	26	2	25	5	5	19	ST38 (ST38 Cplx)
**ED8**	6	19	3	16	11	8	6	ST1727
**ED157**	54	66	17	27	286	4	4	ST3573
**ED166**	83	331	42	44	1	2	2	ST4166

Supplementary table. 3. Accessory genome of each genome group. PFs indicate protein families.

	N° of PFs
Accessory genome of *Daphnia* strains vs Genomes of freshwater ones	1369
Accessory genome of *Daphnia* strains vs Genomes of poultry meat ones	873

Accessory genome of Freshwater vs Genomes of *Daphnia* strains	492
Accessory genome of poultry meat vs Genomes of *Daphnia* strains	1284
Accessory genome of *Daphnia* strains vs Genomes of the other *E. coli* groups	818

Supplementary Table 4: Percentage of the gfp gene found in the gut and carapax samples of dissected *Daphnia*

	ED1-gfp		ED157-gfp	
% carapax	%gut	samples	% carapax	%gut
88.20586	11.79414	1	30.55211	69.44789
66.67754	33.32246	2	36.37496	63.62504
73.28058	26.71942	3	28.15056	71.84944
80.6547	19.3453	4	44.26287	55.73713
74.56091	25.43909	5	22.66621	77.33379
78.94122	21.05878	6	30.60439	69.39561
52.08777	47.91223	7	31.78352	68.21648
53.51601	46.48399	8	15.38194	84.61806
69.41398	30.58602	9	32.33105	67.66895
